# Kaempferol Inhibits Hepatic Stellate Cell Activation by Regulating miR-26b-5p/Jag1 Axis and Notch Pathway

**DOI:** 10.3389/fphar.2022.881855

**Published:** 2022-06-01

**Authors:** Guangyao Zhou, Chunxue Li, Rongrong Zhang, Yating Zhan, Lifan Lin, Zhichao Lang, Qiqi Tao, Jianjian Zheng

**Affiliations:** ^1^ Department of Infectious Diseases, The Second Affiliated Hospital and Yuying Children’s Hospital of Wenzhou Medical University, Wenzhou, China; ^2^ Key Laboratory of Clinical Laboratory Diagnosis and Translational Research of Zhejiang Province, The First Affiliated Hospital of Wenzhou Medical University, Wenzhou, China

**Keywords:** kaempferol, miR-26b-5p, JAG1, hepatic stellate cell, liver fibrosis

## Abstract

Kaempferol, a natural flavonoid molecule, has demonstrated anti-inflammatory, antimicrobial and antioxidant activities. Recent studies have shown the beneficial effects of kaempferol on liver fibrosis. Notch pathway has been reported to be involved in the aberrant activation of hepatic stellate cells (HSCs). However, whether Notch pathway plays a key role in the anti-fibrotic effects of kaempferol is largely unknown. In this study, kaempferol significantly suppressed liver fibrosis in CCl_4_ mice, with reduced collagen deposition as well as restored liver function. *In vitro*, kaempferol enhanced the suppression of HSC activation, with a decrease in *α*-SMA as well as collagen level. It was found that Notch pathway played an important role in kaempferol-reduced the activation of HSCs. Jag1, a ligand of Notch pathway, was obviously inhibited by kaempferol. Overexpression of Jag1 effectively abolished kaempferol-induced HSC inactivation. Furthermore, Jag1 was demonstrated as a target of microRNA-26b-5p (miR-26b-5p). Interestingly, miR-26b-5p inhibitor prevented HSC activation inhibition caused by kaempferol. Further studies indicated that kaempferol inhibited Notch pathway *via* miR-26b-5p and Jag1, leading to HSC inactivation. Collectively, we demonstrate that kaempferol could inhibit HSC activation, at least in part, *via* miR-26b-5p-mediated Jag1 axis and Notch pathway. Kaempferol may serve as a promising drug in the application of treating liver fibrosis.

## Introduction

Liver fibrosis, as a diffuse disease caused by various liver injury, is a common pathological process of chronic liver diseases. Currently, liver fibrosis is mainly induced by hepatitis virus infection or alcoholism, resulting in a rock-ribbed health problem worldwide (Ueha et al., 2012). The main characteristics of liver fibrosis are excessive extracellular matrix (ECM) proteins including type I collagen. In response to sustained inflammatory stimuli and chronic injury, hepatic stellate cells (HSCs) undergo “activation” and transform from store vitamin A cells into proliferating, migrating and contracting myofibroblasts, which is considered as a key event in the development of liver fibrosis (Faraj et al., 2011; Tokunaga et al., 2017). Therefore, effectively down-regulating HSC activation is one of promising therapeutic strategies for ameliorating liver fibrosis.

MicroRNAs (miRNAs) are a class of non-coding RNAs with a length of 20–24 nt (Zeng et al., 2019). In general, miRNAs negatively regulate gene expression *via* targeted mRNA cleavage or translation inhibition at the post-transcriptional level (Elovic et al., 2019). Recent studies have demonstrated that miRNAs participate in cellular biological functions such as proliferation and apoptosis (Kitano and Bloomston, 2016). Deregulated miRNAs are widely found in various human diseases including cancers, which may contribute to the progression of diseases (Sharma et al., 2021). Yang et al. revealed that miR-26b-5p acts as an antifibrotic miRNA in liver fibrosis *via* targeting PDGFR-β (Yang et al., 2019). We previously demonstrated that pinostilbene hydrate-induced HSC inactivation is through miR-17-5p and WIF1 (Zhou et al., 2020). Combined with these, miRNAs play an irreplicable role in the regulation of HSC activation, and targeting liver fibrosis-related miRNAs may be one of the promising treatment strategies.

Kaempferol (3,5,7-trihydroxy-2-(4-hydroxyphenyl)-4H-1-benzopyran-4-one), a natural flavonoid molecule, is widely present in fruits, vegetables and traditional herbs (Kim et al., 2018; Ren et al., 2019). Kaempferol has demonstrated anti-inflammatory, antimicrobial and antioxidant activities (Rho et al., 2011). Recently, Li et al. demonstrated that kaempferol contributes to the suppression of collagen synthesis, proliferation and activation of fibroblasts in hypertrophic scar (HPS) (Li et al., 2016). Interestingly, Xu et al. found that kaempferol-ameliorated liver fibrosis may be *via* activin receptor-like kinase 5 (Xu et al., 2019). However, the potential molecular mechanisms of kaempferol in the regulation of liver fibrosis progression are still largely unknown. Herein, it was found that kaempferol ameliorated the development of liver fibrosis *in vivo* as well as *in vitro*. Kaempferol was shown to suppress the activation of HSCs, at least in part, *via* miR-26b-5p-mediated Notch pathway.

## Materials and Methods

### Materials

**FIGURE 1:**
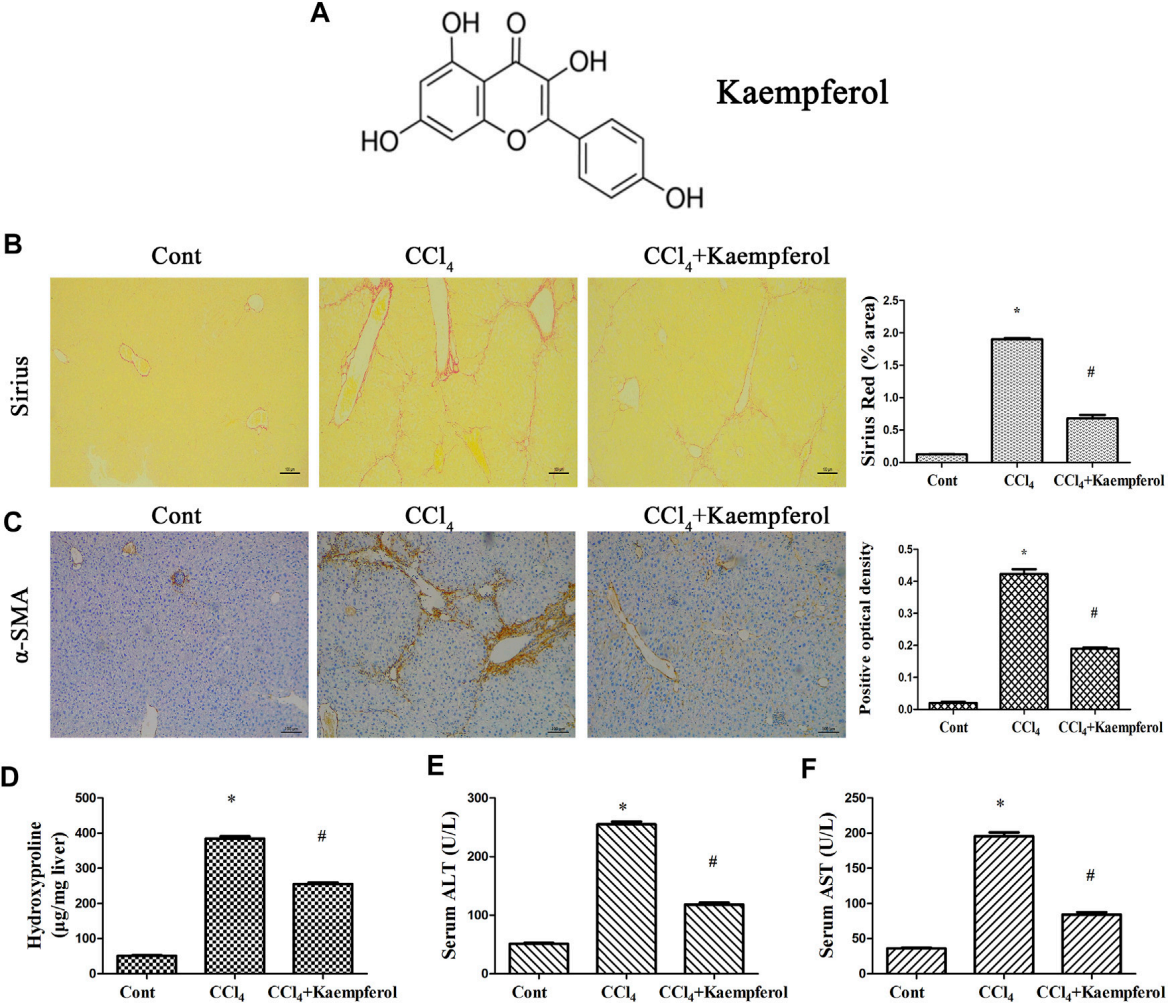
Kaempferol alleviates liver fibrosis caused by CCl_4_ *in vivo*. **(A)** The chemical structure of kaempferol. **(B)** Analysis of Sirius Red staining for collagen deposits. Scale bar, 100 μm. **(C)** Analysis of *α*-SMA immunohistochemistry. Scale bar, 100 μm. **(D)** Hydroxyproline level. **(E)** Serum ALT level. **(F)** Serum AST level. *p < 0.05 compared with the control and ^#^ *p* < 0.05 compared with CCl_4_ group.

MiRNA negative control (miR-NC), miR-26b-5p mimics/inhibitor, adenoviral vectors expressing Jag1 (Ad-Jag1), adenoviral vectors expressing a control scrambled sequence (Ad-Ctrl), adenoviral vectors expressing shRNA against Jag1 (Ad-shJag1) and adenoviral vectors expressing the scrambled shRNA (Ad-shCtrl) were obtained from GenePharma biotechnology (Shanghai, China). The molecular weight and molecular formula of kaempferol (≥97% purity, Sigma, St Louis, MO, United States) are 286.24 and C_15_H_10_O_6_, respectively (Figure 1A).

### Animal Experiments

Liver fibrosis was produced by intraperitoneal injection with carbon tetrachloride (CCl_4_) (Sigma, St Louis, MO, United States) in olive oil (10%, 7 μL/g mice) two times weekly for 4 weeks in 8-week-old C57BL/6 J mice (*n* = 6). Meanwhile, mice in control group were given the same volume of olive oil (*n* = 6). In kaempferol group, mice (*n* = 6) were treated with kaempferol (10 μmol/L, 1 ml) daily *via* intraperitoneal injection during CCl_4_ period (Xu et al., 2019). This project was approved by the University Animal Care and Use Committee of Wenzhou Medical University. Mice, obtained from the Experimental Animal Center of Wenzhou Medical University, were sacrificed under anesthesia after the end of animal experiment. Finally, blood samples and liver tissues were collected for further analysis such as Sirius red staining.

### Immunohistochemistry

Briefly, the tissues were immersed in 4% formalin for fixation, and then the formalin-fixed tissue is degreased and rehydrated. Next, the sections, blocked in 10% BSA, were in the incubation with anti-α-SMA primary antibody at 4°C for at least 12 h. Then, the sections were incubated with a horseradish peroxidase-conjugated goat anti-rabbit IgG secondary antibody for 30 min. Finally, quantitative analysis for *α*-SMA-positive areas was performed under the microscope (Carl Zeiss, Germany).

### Hepatic Hydroxyproline Content

According to the manufacturer’s instructions, the hydroxyproline content in liver tissue was measured using the Hydroxyproline Colorimetric Assay kit (BioVision, San Francisco, CA) and liver weight was used to normalize the relative abundance of hydroxyproline. Briefly, liver tissues were mixed with HCl for homogenization, and then hydrolyzed for 12 h. The supernatant was obtained from the lysate, which was centrifuged at 12,000 g for 10 min at 4°C.

### Primary HSCs Isolation and Culture

According to the previously described enzyme digestion and density gradient centrifugation, isolated primary HSCs were obtained (Chang et al., 2014). The purity of primary HSCs was >98%, which was defined by *α*-SMA and Desmin immunocytochemical staining. Primary 1-day-old HSCs were treated with 10 μM kaempferol or 20 μM curcumin (Cur) for 24 h, respectively. Cells were transfected with 100 nM miR-26b-5p mimic using lipofectamine RNAiMAX.

### Signaling Pathway Assay

The Cignal Finder 10-Pathway Reporter Array (QIAGEN, MA, United States) was performed to determine the kaempferol-related signaling pathways. Attractene (1 μL/well) was distributed into Cignal Finder 10-Pathway Reporter Array plate. Then, primary HSCs cell suspension was diluted in Opti-MEM medium and seeded in each well. Cells were treated with 10 μM kaempferol on the following day. After 24 h of treatment, reverse transfection reagent and Opti-MEM medium were removed. Dual-Glo Luciferase Reagent (75 μL) was then added to each well and plates were incubated for 10 min at room temperature. Finally, firefly and Renilla luciferase activities were measured following the manufacturer’s recommendations.

### Quantitative Real-Time PCR Analysis

Total RNA was isolated from primary HSCs as well as liver tissues using the Tiangen RNA extraction reagent kit. Each sample (1 µg RNA) was reversely transcribed into complementary DNA (cDNA) using a reverse-transcription (RT) reagent kit (Takara Biotechnology Co., Ltd., Dalian, China). Then, Real-time PCR was performed using SYBR Premix ExTaq (Takara). As shown in Supplementary Table S1, the primers of *α*-SMA, alpha-1(I) collagen (Col1A1), hes family bHLH transcription factor 1 (Hes1), Hes5, Notch1-4, Delta-like 1 (Dll1), Dll3, Dll4, Jagged 1 (Jag1), Jag2 and GAPDH were designed. TaqMan MicroRNA Assays (Applied Biosystems, Foster City, CA) were performed to detect 10 miRNAs expressions. GAPDH and U6 were used as endogenous controls for mRNAs and for miRNAs, respectively.

### Western Blot Analysis

The proteins from primary HSCs and liver tissues were extracted using RIPA extraction buffer. The protein samples of interested were separated by 10% SDS-PAGE electrophoresis, and then transferred to PVDF membranes. The primary anti-Jag1, anti-α-SMA, type I collagen and anti-GAPDH (an internal control) were added in PVDF membranes and incubated overnight at 4°C. Then, the second antibody was added and incubated at room temperature for 1 h.

### Immunofluorescence

In brief, cells were fixed in 4% paraformaldehyde for 20 min at room temperature, and then permeabilized with PBS-0.5% Triton X-100 for 15 min. Subsequently, blocking with 5% BSA in PBS for 1 h at 37°C and incubated with the following primary antibodies overnight at 4°C in a humidified chamber: anti-α-smooth muscle actin (α-SMA) (1:100) and anti-type I collagen (1:100). On the next day, the cells were incubated with secondary antibodies conjugated with Alexa Fluor 488 or 546 (Invitrogen), respectively. Nuclei were stained using 4,6-diamidino-2-phenylindole (DAPI), and cells were observed using a Nikon Eclipse Ti-E inverted fluorescence microscope (Nikon, Tokyo, Japan).

### 5-Ethyny-2ʼ-Deoxyuridine Assay

EdU assay was used to detect cell proliferation. After kaempferol or Cur treatment, cells were labeled with EdU for 12 h, and EdU^+^ population represented the proliferating cell population.

### Luciferase Reporter Assay

As described previously, miR-26b-5p or miR-NC was co-transfected with pmirGLO-Jag1 into HEK293T cells by lipofectamine-mediated gene transfer (Yu et al., 2017). After 24 h, the luciferase signals were detected using the Dual-Luciferase^®^ Reporter Assay System (Promega). Firefly luciferase intensity was normalized over Renilla luciferase signal.

### Statistical Analysis

Data were presented as the means ± SD. One-way analysis of variance (one-way ANOVA) was employed for multiple groups. Differences between two groups were compared using student’s t-test. *p* < 0.05 was considered significant. The SPSS software was used for all statistical analyses (version 16.0; SPSS, Chicago, IL).

## Results

### Kaempferol Alleviates Liver Fibrosis Caused by CCl_4_ *in vivo*

The roles of kaempferol were firstly examined in CCl_4_-induced mouse liver fibrosis model. As shown by Sirius red staining and immunohistochemical analysis, both collagen deposition and *α*-SMA expression were induced by CCl_4_, respectively (Figures 1B,C). Hydroxyproline assays further confirmed enhanced collagen level by CCl_4_ (Figure 1D). Liver function of CCl_4_ mice was shown to be injured, associated with increased ALT and AST (Figures 1E,F). Notably, kaempferol significantly inhibited CCl_4_-caused collagen (Figures 1B,D). Kaempferol resulted in the reduction in *α*-SMA expression (Figure 1C). Furthermore, kaempferol ameliorated liver function (Figures 1E,F). Our results demonstrate that kaempferol ameliorates CCl_4_-induced liver fibrosis *in vivo*.

### Kaempferol Decreases the Activation of HSCs

**FIGURE 2:**
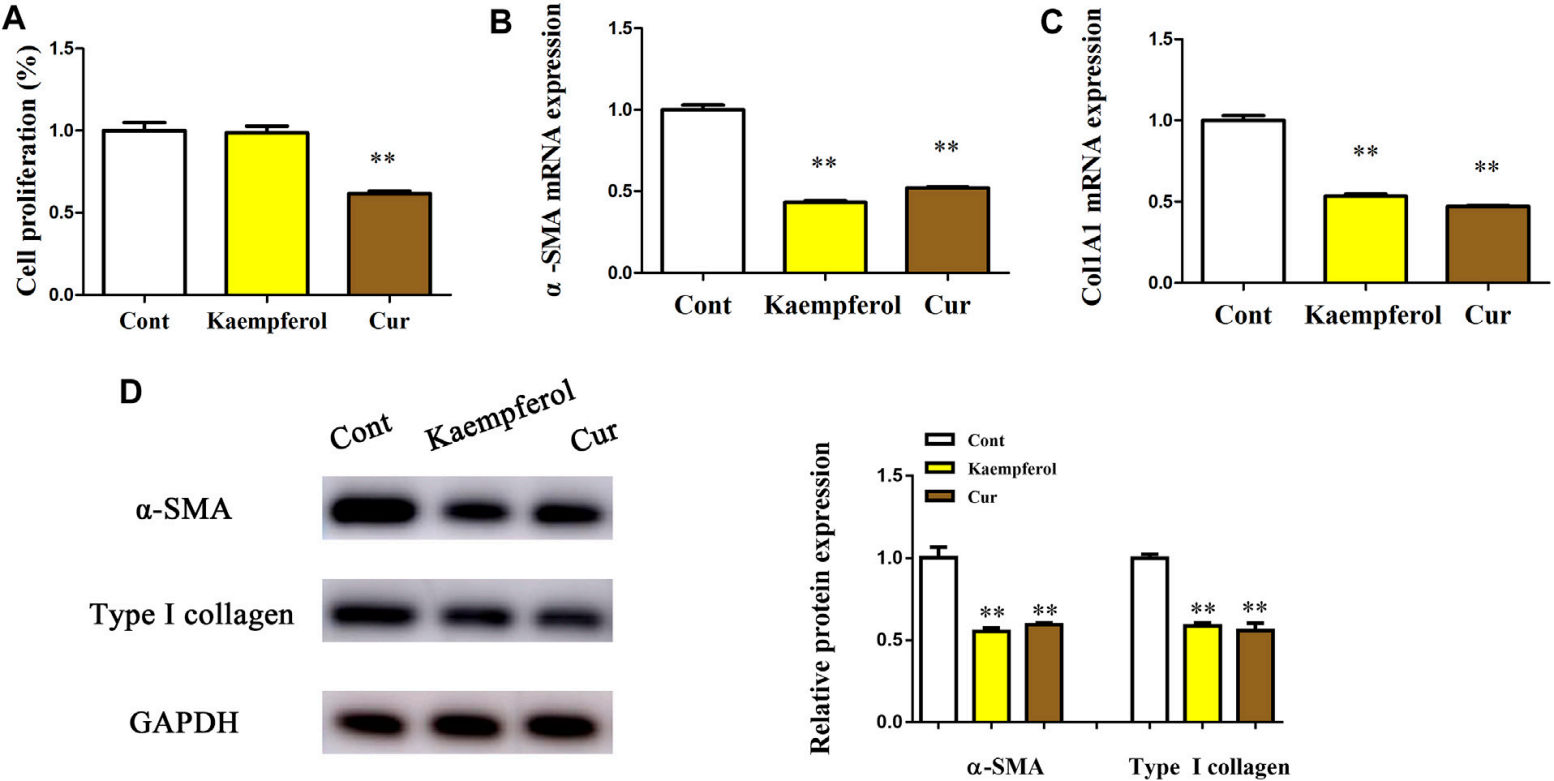
Effects of kaempferol on HSC proliferation and activation. Primary 1-day-old HSCs were treated with 10 μM kaempferol for 24 h. **(A)** Cell proliferation. **(B)** *α*-SMA mRNA. **(C)** Col1A1 mRNA. **(D)** Levels of *α*-SMA protein and type I collagen. **p < 0.01 compared with the control.

Whether kaempferol contributed to the suppression of HSC activation was subsequently investigated. Cur, which has been demonstrated to suppress HSC activation, was used as a positive control (Zhang et al., 2014). Obviously, Cur inhibited HSC proliferation, HSC transdifferentiation as well as collagen expression. Our results showed that Cur induced a significant reduction in HSC proliferation, HSC transdifferentiation and collagen level (Figures 2A–C). As shown by Edu assays, kaempferol caused no significant change in cell proliferation (Figure 2A). Kaempferol obviously inhibited HSC transdifferentiation, with reduced *α*-SMA mRNA level (Figure 2B). Moreover, kaempferol resulted in the suppression of Col1A1 mRNA level (Figure 2C). Accordingly, immunoblot results indicated that both *α*-SMA protein and type I collagen were inhibited by kaempferol (Figure 2D). Overall, the above data suggest that kaempferol plays an inhibitory role in the activation of HSCs.

### Kaempferol Promotes HSC Activation Inhibition *Via* Down-Regulating Jag1

**FIGURE 3:**
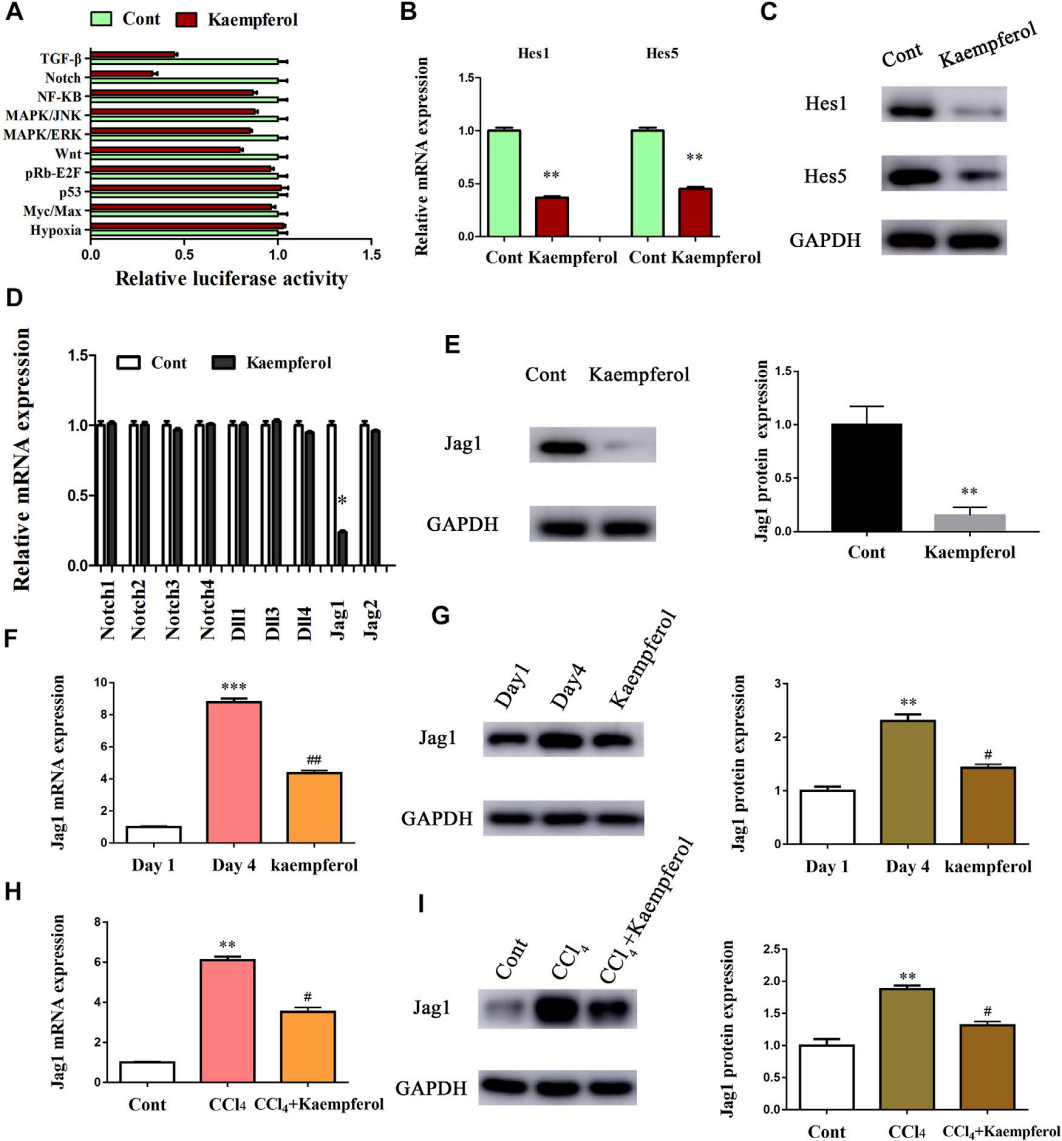
Kaempferol inhibits HSC activation through Notch pathway. Primary 1-day-old HSCs were treated with 10 μM kaempferol for 24 h. **(A)** Signaling pathway reporter array was performed to search for kaempferol-related pathways. mRNA **(B)** and protein **(C)** expressions of Hes1 and Hes5. Three experiments with similar outcome. **(D)** mRNA expressions of Notch receptors and ligands. **(E)** Jag1 protein level in kaempferol-treated cells. mRNA **(F)** and protein **(G)** expressions of Jag1 in primary HSCs during cell culture. mRNA **(H)** and protein **(I)** expressions of Jag1 in CCl_4_ mice. **p < 0.01, ***p < 0.01 compared with the control. ^*#*^ *p* < 0.05, ^#*#*^ *p* < 0.01 compared with the Day4 or CCl_4_ group.

**FIGURE 4:**
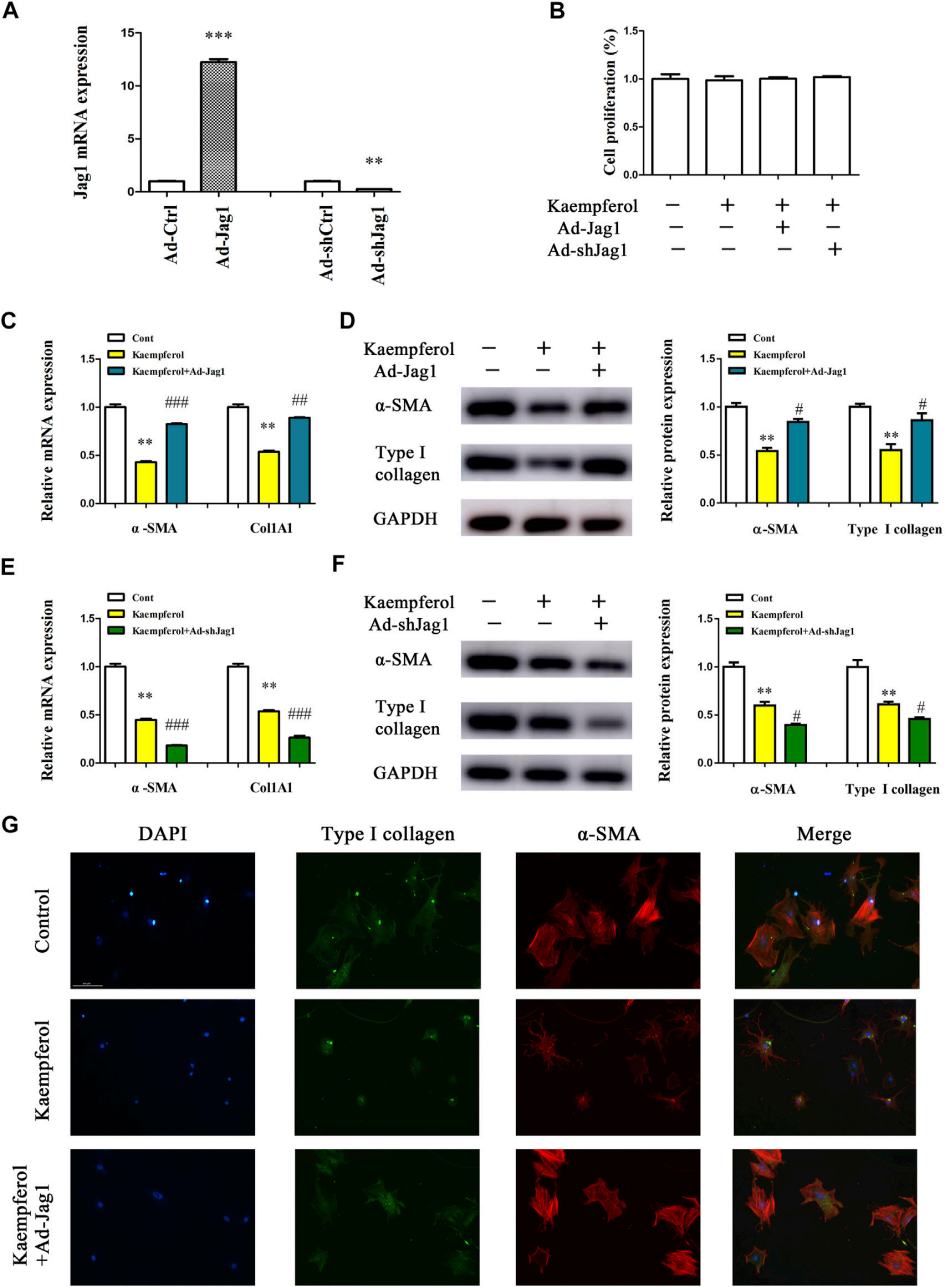
Kaempferol inhibits HSC activation *via* down-regulating Jag1. Primary 1-day-old HSCs were treated with 10 μM kaempferol for 24 h and then transduced with Ad-Jag1/Ad-shJag1 for additional 24 h **(A)** Jag1 expression. **(B)** Cell proliferation. mRNA **(C)** and protein **(D)** expressions of *α*-SMA and Col1A1 in cells with Jag1 overexpression. mRNA **(E)** and protein **(F)** expressions of *α*-SMA and Col1A1 in cells with Jag1 knockdown. **(G)** Immunofluorescence staining for *α*-SMA (red) and type I collagen (green). DAPI stained nuclei blue. **p < 0.01, ***p < 0.01 compared with the control, and ^#^ *p* < 0.05, ^##^ *p* < 0.01, ^###^ *p* < 0.001 compared with kaempferol group.

Next, the underlying molecular mechanism of kaempferol in the regulation of HSC activation was examined. A pathway reporter array was used to rapidly identify the relevant pathways in cells with kaempferol. Clearly, kaempferol resulted in an obvious suppression in six signaling pathways including TGF-β, Notch, NF-κB, MAPK/JNK, MAPK/ERK and Wnt, with the lowest expression in Notch pathway (Figure 3A). Our results suggest that the Notch pathway may take a part in the effects of kaempferol-inhibited HSC activation (Figure 3A). Subsequently, Hes1 and Hes5, which are the downstream of the Notch pathway, were examined in cells after kaempferol treatment. In comparison with non-treated cells, kaempferol induced a significant reduction in the mRNA expressions of Hes1 and Hes5 (Figure 3B). Accordingly, the protein levels of Hes1 and Hes5 were down-regulated by kaempferol (Figure 3C). Generally, Notch signaling is initiated by the activated Notch receptors and ligands (Kovall et al., 2017). In kaempferol-treated cells, the expressions of Notch receptors as well as ligands were measured. Our results showed that the level of Jag1 was reduced after kaempferol treatment, whereas others not (Figure 3D). Consistent with the mRNA result, Jag1 protein was also decreased in kaempferol-treated cells (Figure 3E), indicating that Jag1 may participate in the effects of kaempferol on HSC activation inhibition. Thus, Jag1 expression was detected *in vitro* and *in vivo* during HSC activation. It was found that Jag1 expression was obviously enhanced in primary HSCs during cell culture, which was inhibited by kaempferol (Figures 3F,G). Similar with the results *in vitro*, Jag1 expression was up-regulated in CCl_4_ mice, which was also suppressed by kaempferol (Figures 3H,I). Whether Jag1 plays a key role in the inhibitory effects of kaempferol on the activation of HSCs was further explored. As shown in Figure 4A and Supplementary Figure S1, overexpression of Jag1 was found in Ad-Jag1 group, while loss of Jag1 was shown in Ad-shJag1 group. Compared with kaempferol group, the change of proliferative activity between Ad-Jag1 and Ad-shJag1 group was no significant (Figure 4B). Interestingly, overexpression of Jag1 blocked down kaempferol-inhibited *α*-SMA and Col1A1 (Figures 4C,D). On the contrary, loss of Jag1 enhanced the inhibition of *α*-SMA and Col1A1 in kaempferol-treated cells (Figures 4E,F). Additionally, analysis of immunofluorescence confirmed that kaempferol-reduced type I collagen (green fluorescence) and *α*-SMA (red fluorescence) was blocked down by Ad-Jag1 (Figure 4G and Supplementary Figure S2A). These data suggest that the inhibitory role of kaempferol in the regulation of HSC activation is *via* suppressing Jag1, leading to the inactivation of Notch pathway.

### Jag1 is a Target of miR-26b-5p

**FIGURE 5:**
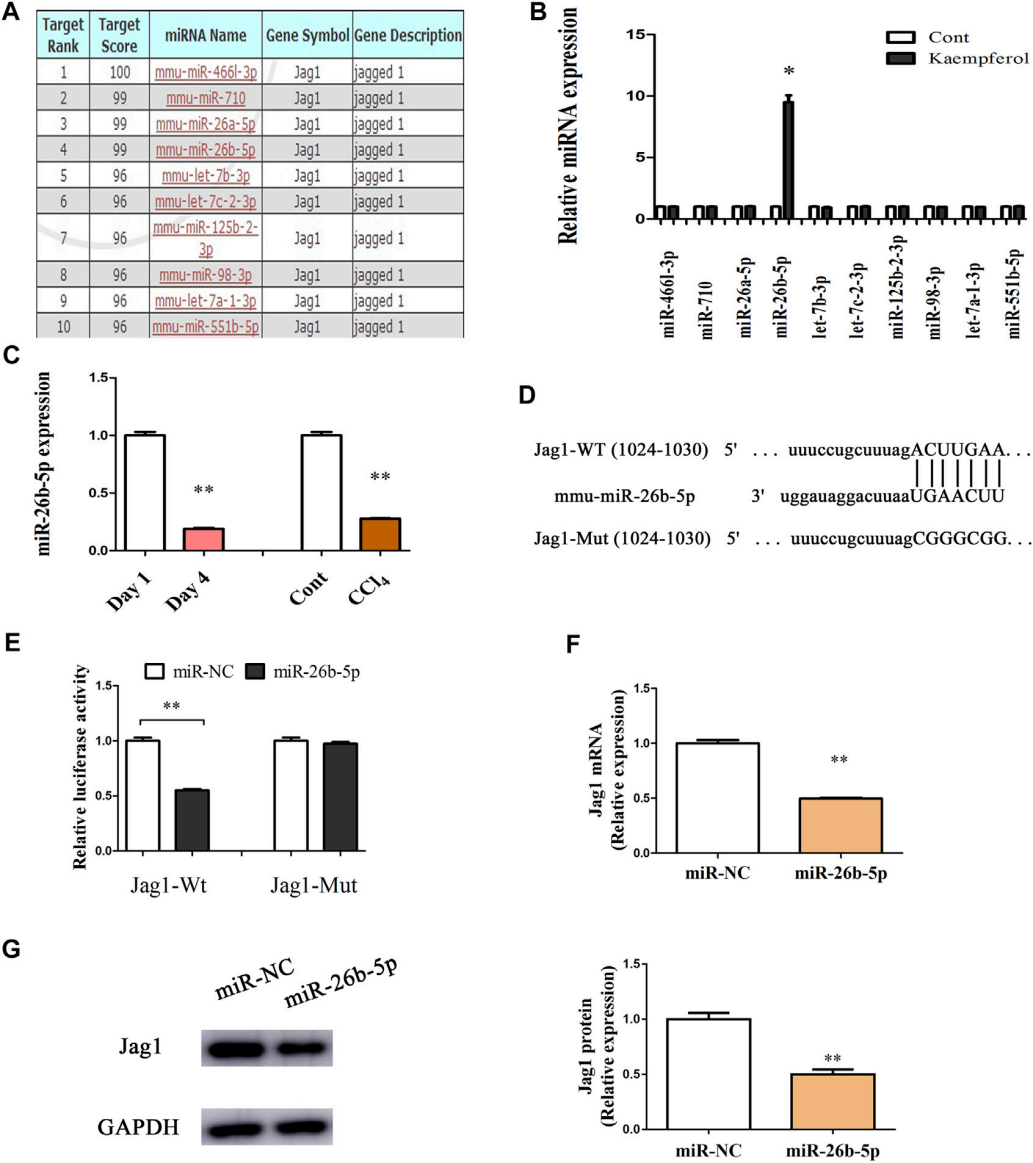
Jag1 is a target of miR-26b-5p. **(A)** 10 predicted miRNAs with the highest target score were shown in bioinformatics analysis (miRDB). **(B)** miR-26b-5p was enhanced in cells with kaempferol. **(C)** miR-26b-5p level *in vitro* and *in vivo*. **(D)** miR-26b-5p-biding sites in the 3′UTR of Jag1 mRNA based on miRDB software. **(E)** Relative luciferase activities of luciferase reporters bearing wild-type or mutant Jag1 were analyzed 48 h following transfection with the indicated miR-26b-5p mimics or miR-NC in HEK293T cells. Jag1 mRNA **(F)** and protein **(G)** expression in cells with miR-26b-5p. *p < 0.05, **p < 0.01 compared with the control.

Recently, miRNA-mediated Jag1 expression has been found in various human diseases (Zhang et al., 2020a; Zhang et al., 2020b). Herein, bioinformatics analysis (miRDB) was performed to identify the potential miRNAs targeting Jag1, and 10 miRNAs with the highest target score were shown in Figure 5A. Interestingly, in kaempferol-treated cells relative to the untreated cells, only miR-26b-5p was significantly up-regulated, whereas other predicted miRNAs not (Figure 5B). Therefore, miR-26b-5p was selected for the next experiments. Both *in vitro* and *in vivo*, the levels of miR-26b-5p were examined during liver fibrosis. Our results showed that reduced miR-26b-5p was found in primary HSCs at Day 4 as well as CCl_4_ mice (Figure 5C), indicating that the miR-26b-5p was negatively correlated with the Jag1. Thus, the Jag1 target region was cloned in the pmirGLO plasmid to generate a Jag1 pmirGLO luciferase reporter containing the miR-26b-5p binding sites (Figure 5D). Luciferase activity assays were performed to determine whether Jag1 is a target of miR-26b-5p. Clearly, miR-26b-5p induced a significant reduction in luciferase activity driven by the wild-type 3′UTR of Jag1, and there was no significant difference in mutant-type 3′UTR of Jag1 (Figure 5E). In primary HSCs, miR-26b-5p overexpression down-regulated Jag1 mRNA as well as protein expression (Figures 5F,G). Our data suggest that miR-26b-5p induced by kaempferol targets Jag1.

### Kaempferol Suppresses HSC Activation Through miR-26b-5p-Mediated Jag1 Axis

**FIGURE 6:**
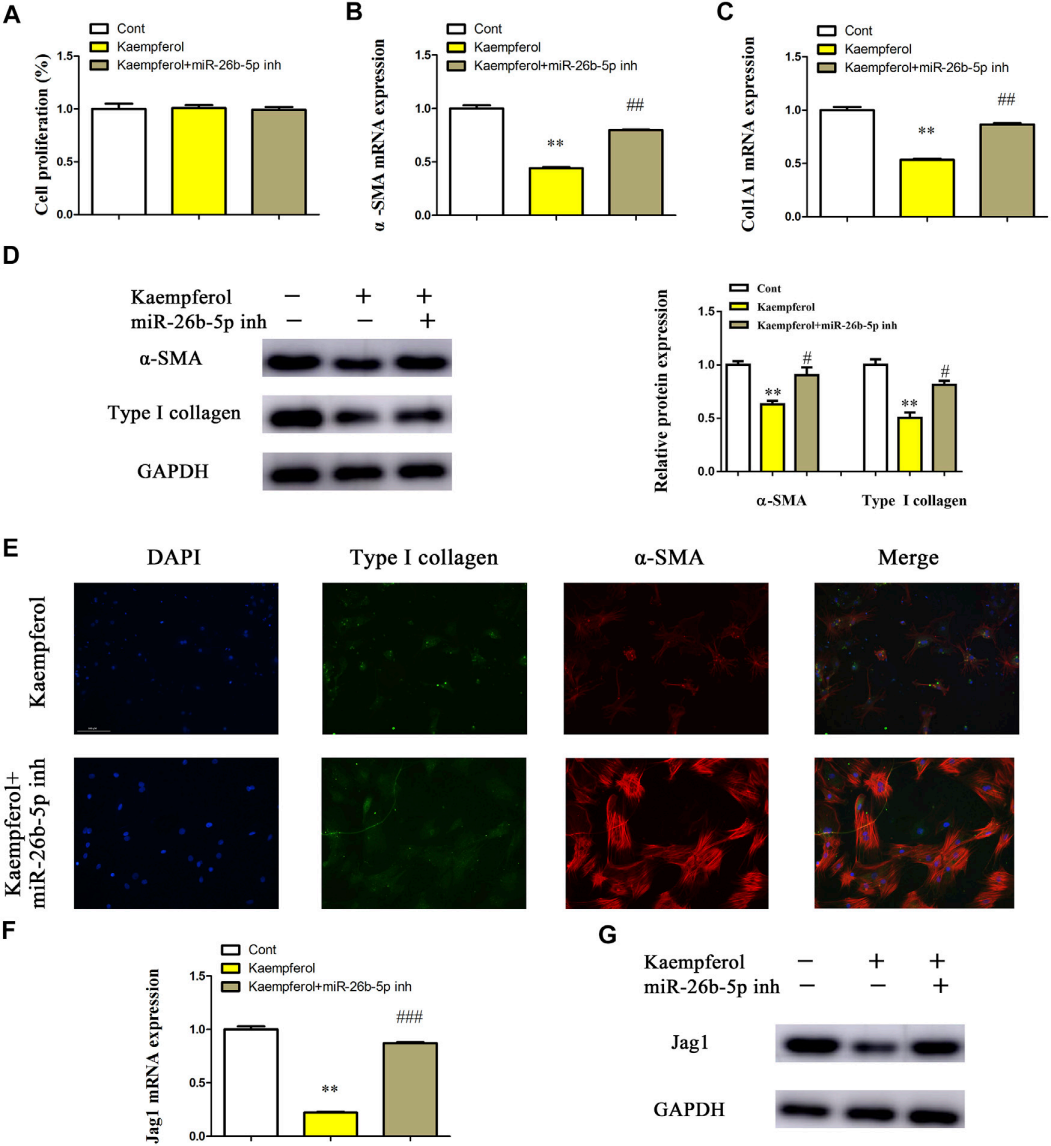
Kaempferol suppresses HSC activation through miR-26b-5p-mediated Jag1 axis. Primary 1-day-old HSCs were treated with 10 μM kaempferol for 24 h and then transfected with 100 nM miR-26b-5p inhibitor for additional 24 h. **(A)** Cell proliferation. **(B)** *α*-SMA mRNA level. **(C)** Col1A1 mRNA level. **(D)** Protein expressions of *α*-SMA and Type I collagen in kaempferol-treated HSCs after miR-26b-5p inhibitor treatment. **(E)** Immunofluorescence staining for *α*-SMA (red) and type I collagen (green). DAPI stained nuclei blue. **(F,G)** Jag1 mRNA and protein were detected in kaempferol-treated HSCs after miR-26b-5p inhibitor treatment. Three experiments with similar outcome. **p < 0.01 compared with the control, and ^#^ *p* < 0.05, ^##^ *p* < 0.01, ^###^ *p* < 0.001 compared with kaempferol group.

Whether miR-26b-5p plays a crucial role in the inhibitory effects of kaempferol on HSC activation was further investigated. After transfection with miR-26-5p inhibitor, the proliferation of kaempferol-treated cells was detected by EdU analysis. In line with our previous results, miR-26-5p had no effect on cell proliferation in comparison with kaempferol group (Figure 6A). Notably, loss of miR-26-5p blocked down kaempferol-reduced *α*-SMA mRNA expression (Figure 6B). Likewise, miR-26-5p inhibitor significantly suppressed kaempferol-inhibited Col1A1 expression (Figure 6C). Accordingly, the results of immunoblot indicated that miR-26-5p inhibitor restored *α*-SMA and type I collagen in cells with kaempferol (Figure 6D). Results of immunofluorescence further revealed that miR-26-5p inhibitor contributed to the restoration of *α*-SMA and type I collagen in kaempferol-treated cells (Figure 6E and Supplementary Figure S2B). Moreover, kaempferol-inhibited Jag1 was restored by miR-26b-5p inhibitor (Figures 6F,G). Therefore, kaempferol suppresses HSC activation, at least in part, through miR-26b-5p-mediated Jag1 axis.

## Discussion

Kaempferol has been demonstrated to have many properties in various human diseases. One of the well-known properties of kaempferol is anti-inflammation. Previously, Gong et al. found that kaempferol inhibits the progression of airway inflammation *via* down-regulating NF-κB pathway (Gong et al., 2012). Besides, increasing studies have shown the beneficial effects of kaempferol on cancers including hepatocellular carcinoma (Guo et al., 2016). Recently, it has been reported that kaempferol has beneficial effects against fibroproliferative disorders including liver fibrosis and HPS. For instance, kaempferol suppresses TGF-β/Smads signalling in liver fibrosis *via* directly binding to the catalytic region of ALK5 (Xu et al., 2019). However, the detailed mechanisms of kaempferol against liver fibrosis are still unknown. In this study, kaempferol was found to have beneficial effects against liver fibrosis both *in vivo* and *in vitro*, associated with the downregulated expressions of *α*-SMA level and collagen. In addition, CCl_4_-induced serum ALT/AST level was inhibited by kaempferol, indicating that kaempferol treatment contributes to the restoration of liver function during liver fibrosis. Further studies confirmed that miR-26b-5p-mediated Notch pathway was highly correlated with the beneficial effects of kaempferol on liver fibrosis. Owing to the restoration of miR-26b-5p, Jag1 level was inhibited, leading to the suppression of Notch pathway, which finally resulted in the inhibition of HSC activation. Our results demonstrate that kaempferol inhibits HSC activation, at least in part, *via* miR-26b-5p/Jag1 axis, and this is a first report.

The Notch pathway, a highly conserved signalling, has been shown to play critical roles in animal development, tissue homeostasis and human diseases (Artavanis-Tsakonas and Muskavitch, 2010; Siebel and Lendahl, 2017). There are four Notch receptors (NOTCH1-4) and five canonical ligands (DLL1, DLL3, DLL4, JAG1 and JAG2) in mammals. Notch pathway is essential to various cellular processes such as cell fate specification, proliferation and apoptosis (Bray, 2016). Emerging evidence has shown that aberrant Notch pathway activation contributes to the initiation and progression of cancers (Villanueva et al., 2012). Activated Notch pathway is also involved in human fibrotic diseases including liver fibrosis (Geisler and Strazzabosco, 2015). Recent studies have shown that during HSC activation, Jag1 expression is enhanced in rat HSCs (Sawitza et al., 2009). Tang et al. further confirmed that loss of Jag1 pathway results in the suppression of HSC activation (Tang et al., 2017). Herein, it was found that Notch pathway was significantly inhibited by kaempferol, associated with reduced Hes1 and Hes5. Similar with the previous studies, Jag1 expression was enhanced during liver fibrosis, which was obviously down-regulated by kaempferol. In addition, Jag1 overexpression blocked down the effects of kaempferol on HSC activation inhibition, whereas loss of Jag1 had opposite effects. In sum, kaempferol inhibits HSC activation *via* Jag1-mediated Notch pathway.

MiR-26b-5p, a member of miR-26 family, functions as a human tumor suppressor. For example, miR-26b-5p, down-regulated in breast cancer, induces breast cancer cell apoptosis *via* targeting SLC7A11 (Liu et al., 2011). Importantly, miR-26b-5p is involved in a series of pathophysiological processes such as angiogenesis and inflammation. Increasing evidence has shown that miR-26b-5p could act as a fibrosis suppressor in fibrotic diseases including liver fibrosis (Chen et al., 2017; Yang et al., 2019). At the present study, we examined 10 predicted miRNAs in HSCs after kaempferol treatment, which may interact with Jag1. In comparison with the untreated cells, miR-26b-5p was induced by kaempferol, whereas other miRNAs not. miR-26b-5p was shown to be decreased in CCl_4_ mice and primary HSC, whereas enhanced Jag1 was found in liver fibrosis. Further studies confirmed that Jag1 was a target of miR-26b-5p. Interestingly, miR-26-5p inhibitor could alleviate the effects of kaempferol on HSC activation inhibition. Kaempferol-inhibited Jag1 protein was also restored by loss of miR-26b-5p. Taken together, kaempferol inhibited HSC activation, at least in part, *via* miR-26b-5p-mediated Jag1 pathway. However, there were also many limitations in this study. The detailed mechanism of the regulation of kaempferol in miR-26b-5p is still unknown, and further studies are needed in future.

## Conclusion

In conclusion, our results demonstrate that kaempferol could inhibit HSC activation *via* miR-26b-5p/Jag1 axis and Notch pathway. We also provide a new insight of the critical roles of miR-26b-5p-mediated Jag1 in liver fibrosis. Kaempferol may be a novel candidate drug for the treatment of liver fibrosis.

## Data Availability

The original contributions presented in the study are included in the article/Supplementary Material, further inquiries can be directed to the corresponding author.

## Abbreviations

CCl4, carbon tetrachloride; Col1A1, alpha-1(I) collagen; Cur, curcumin; Dll1, Delta-like 1; ECM, extracellular matrix; EdU, 5-Ethyny-2ʼ-Deoxyuridine; Hes1, hes family bHLH transcription factor 1; HPS, hypertrophic scar; HSCs, hepatic stellate cells; Jag1, Jagged 1; miRNAs, microRNAs; miR-NC, miRNA negative control; qRT-PCR, quantitative real-time PCR; α-SMA, α-smooth muscle actin.
