# Supplementary material for: Kaempferol Inhibits Hepatic Stellate Cell Activation by Regulating miR-26b-5p/Jag1 Axis and Notch Pathway
Source: Front Pharmacol. 2022 Jun 1;13:881855. doi: 10.3389/fphar.2022.881855 (PMC9198265; doi:10.3389/fphar.2022.881855)
Supplement: Supplementary file 1 [file Table1.DOCX]

**Table S1 Primer sequences**

| Gene | Forward sequence | Reverse sequence |
| --- | --- | --- |
| α-SMA | 5'-GCCATCTTTCATTGGGATGGA-3' | 5'-CCCCTGACAGGACGTTGTTA-3' |
| Col1A1 | 5'-CGATGGATTCCCGTTCGAGT-3' | 5'-GAGGCCTCGGTGGACATTAG-3' |
| Hes1 | 5'-CAACACGACACCGGACAAAC-3' | 5'-GGAATGCCGGGAGCTATCTT-3' |
| Hes5 | 5'-AGAGCTCCAGCTGCGGAA-3' | 5'-AGCTGCTCTATGCTGCTGTT-3' |
| Notch1 | 5'-CACCAGGGTGGTCAGGAAAA-3' | 5'-GGGCAGCGACAGATGTATGA-3' |
| Notch2 | 5'-CCGTGGGGCTGAAAAATCTC-3' | 5'-GGGTCATCTTCCGACAGCAA-3' |
| Notch3 | 5'-ACTCCTCCTCAGGGAGATGC-3' | 5'-GTGGGGTGAAGCCATCAGG-3' |
| Notch4 | 5'-ACAAGGGATCTGCCAGTGTG-3' | 5'-CAGGTGCAGGAGAAGTGAGG-3' |
| Dll1 | 5'-CGGACCCCAATTCCCCATTT-3' | 5'-TGGTTTTGTCTTAAGCTTCTCTGG-3' |
| Dll3 | 5'-GGGCAGCTGTAGTGAAACCT-3' | 5'-CTTCACCGCCAACACACAAG-3' |
| Dll4 | 5'-AATGGTGGCAGCTGTAAGGACC-3' | 5'-GCTGGCCATAGTAGCCTGG-3' |
| Jag1 | 5'-CCTGCGAGCCAAGGTGTG-3' | 5'-CTCCACCACAACAGTTCCCA-3' |
| Jag2 | 5'-GCCTGGCCGCGTTCTTT-3' | 5'-AGCTCCTCATCTGGAGTGGT-3' |
