# Supplementary figures and images for: Kaempferol Inhibits Hepatic Stellate Cell Activation by Regulating miR-26b-5p/Jag1 Axis and Notch Pathway
Source: Front Pharmacol. 2022 Jun 1;13:881855. doi: 10.3389/fphar.2022.881855 (PMC9198265; doi:10.3389/fphar.2022.881855)

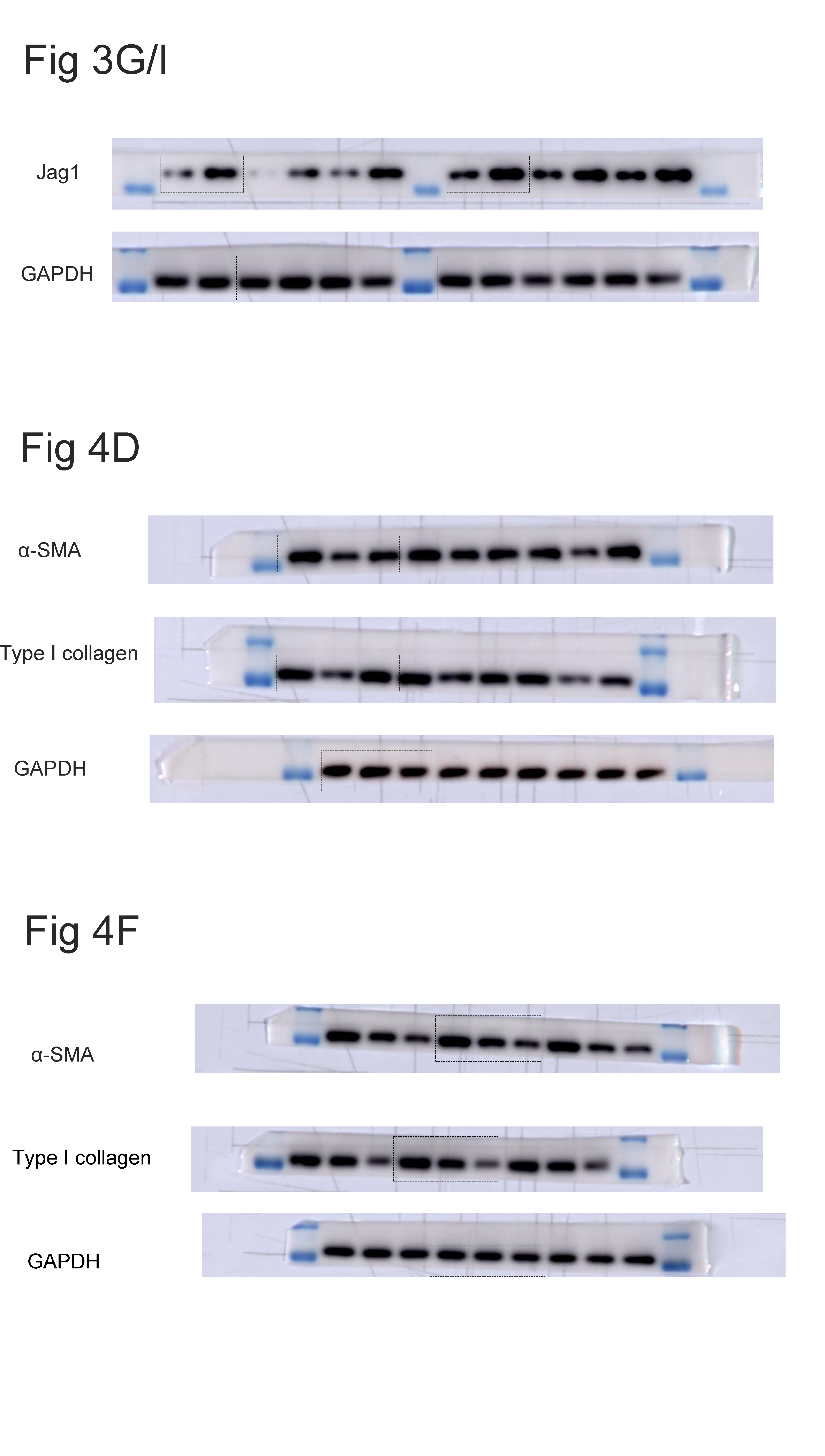

Supplement: Supplementary file 2 [file DataSheet1.ZIP › wb original data/wb-2.tif]
