# Supplementary material for: Kaempferol Inhibits Hepatic Stellate Cell Activation by Regulating miR-26b-5p/Jag1 Axis and Notch Pathway
Source: Front Pharmacol. 2022 Jun 1;13:881855. doi: 10.3389/fphar.2022.881855 (PMC9198265; doi:10.3389/fphar.2022.881855)
Supplement: Supplementary file 3 [file Table2.DOCX]

**Supporting information**


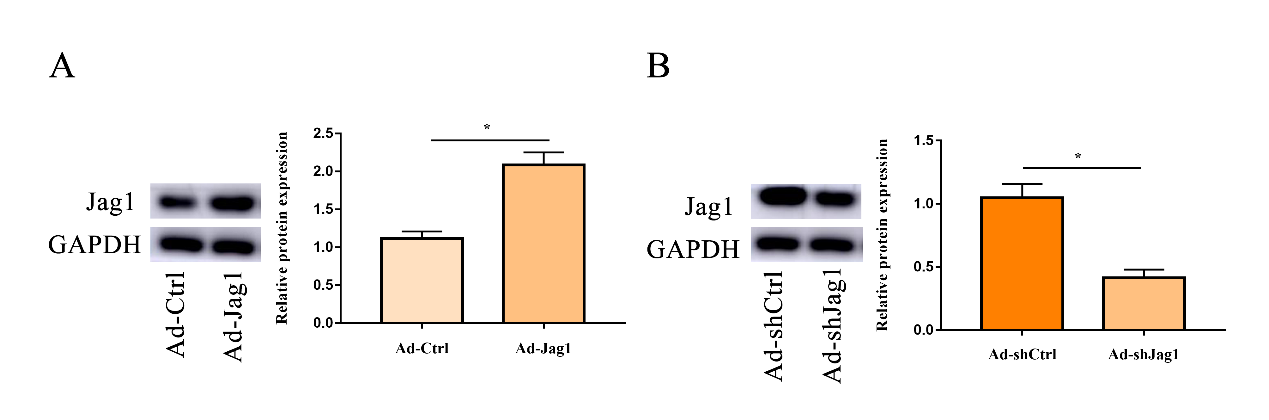


Fig.S1 The effect of Ad-Jag1 and Ad-shJag1 on protein levels. (A)The expression of Jag in Ad-Ctrl and Ad-Jag1. (B) The expression of Jag in Ad-shCtrl and Ad-shJag1. **P*<0.05.


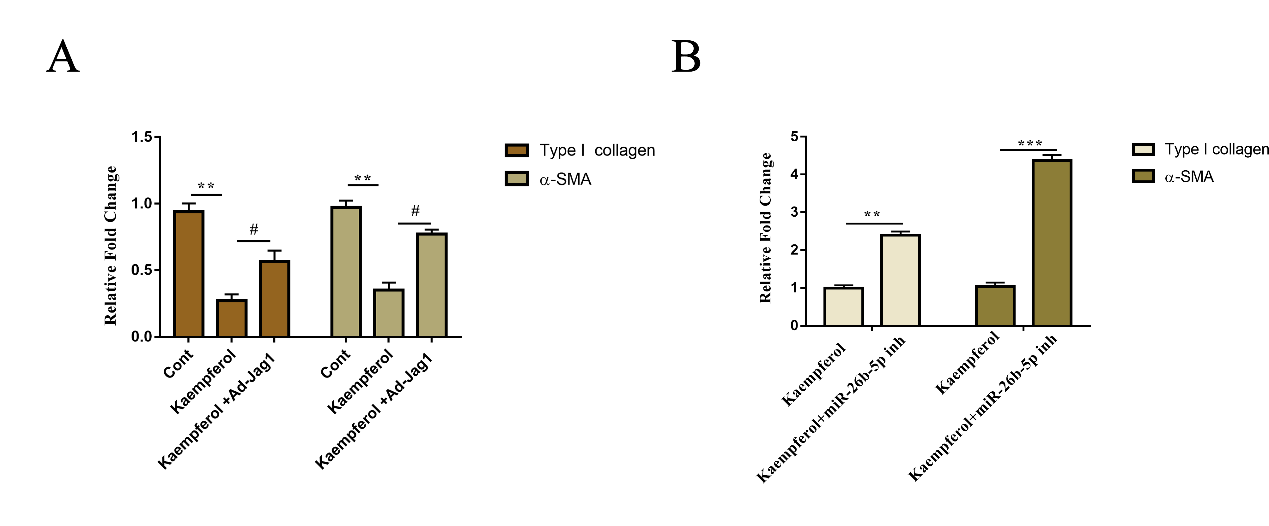


Fig.S2 The quantification and statistical analysis of immunofluorescence. (A) The results of quantification and statistical analysis in Fig.4G. (B) The results of quantification and statistical analysis in Fig.6E. ***P*<0.01, ****P*<0.001 and ^#^*P*<0.05.
